# The role of adjunctive therapies in the management of recurrent rhinosinusitis: a meta-analytical approach

**DOI:** 10.3389/fpubh.2026.1768433

**Published:** 2026-04-22

**Authors:** Lingjuan Nie, Caiping Xu, Yu Liu, Fangxing Wu

**Affiliations:** 1 Department of Otolaryngology, Taizhou Traditional Chinese Medicine Hospital, Taizhou, Zhejiang, China; 2 Department of Otolaryngology, Wujin Hospital of Traditional Chinese Medicine, Changzhou, China; 3 Tongde Hospital of Zhejiang Province, Hangzhou, China

**Keywords:** adjunctive therapy, endoscopic sinus surgery, meta-analysis, nasal irrigation, recurrent rhinosinusitis, steroid-eluting implants

## Abstract

**Background:**

Recurrent and chronic rhinosinusitis remain challenging conditions with high recurrence rates despite advances in medical and surgical management. Adjunctive therapies ranging from nasal irrigation and physiotherapy to steroid-eluting implants and dental interventions are increasingly used to enhance treatment outcomes. This meta-analysis aimed to evaluate the effectiveness of adjunctive therapies in improving clinical and radiological outcomes in patients with recurrent rhinosinusitis.

**Methods:**

A comprehensive literature search was conducted across PubMed, Scopus, ScienceDirect, Google Scholar, and the Consensus Academic Database for studies published between 2015 and 2025. Eligible studies included randomized controlled trials and observational research evaluating adjunctive therapies combined with standard rhinosinusitis treatment. Data extraction and quality assessment followed PRISMA 2020 guidelines using the Cochrane Risk of Bias Tool (RoB 2) and Newcastle Ottawa Scale. Quantitative and qualitative syntheses were performed, with effect sizes expressed as mean differences and 95% confidence intervals.

**Results:**

Nine studies comprising a total of 1,608 patients were included. Adjunctive therapies demonstrated a 30–50% improvement in symptom scores (SNOT-22, RSDI) and a significant reduction in recurrence rates compared to conventional treatment alone. Higher improvement rates were reported in studies evaluating steroid-eluting implants and concurrent dental interventions, which achieved over 90% post-operative success rates. Non-surgical modalities such as nasal irrigation and sinus physiotherapy also yielded meaningful symptomatic relief and improved mucociliary clearance. Heterogeneity was moderate (*I*^2^ < 50%), and no significant publication bias was detected in the funnel plot analysis.

**Conclusion:**

Adjunctive therapies significantly improve symptom control, reduce recurrence, and enhance post-operative outcomes in recurrent rhinosinusitis. Integrating these modalities into standard treatment protocols supports a multimodal, patient-centered approach to disease management. Future multicentric randomized controlled trials with standardized intervention protocols are recommended to confirm and refine these findings.

## 1 Introduction

Chronic and recurrent rhinosinusitis represent significant and often debilitating conditions within otolaryngology practice worldwide (1). According to the European Position Paper on Rhinosinusitis and Nasal Polyps (EPOS 2020) and the 2025 update of the American Academy of Otolaryngology–Head and Neck Surgery (AAO–HNS) Clinical Practice Guideline (2, 3). Chronic rhinosinusitis is defined as inflammation of the nasal and paranasal sinus mucosa persisting for more than 12 weeks, accompanied by objective evidence of mucosal disease (3). Recurrent rhinosinusitis is characterized by repeated symptomatic episodes following appropriate medical or surgical management. Despite advances in diagnostic criteria and standardized treatment algorithms, recurrence and incomplete symptom resolution remain frequent clinical challenges (4).

The persistence and recurrence of disease are increasingly understood as multifactorial processes (5, 6). Impaired mucociliary clearance, chronic inflammatory activation, microbial biofilm formation, allergic comorbidities, and odontogenic infection have all been implicated in sustaining mucosal inflammation and therapeutic resistance (7). These overlapping mechanisms suggest that conventional monotherapy, whether medical or surgical, may not sufficiently address the complex pathophysiology underlying recurrent disease (8). Consequently, attention has shifted toward adjunctive therapeutic strategies that target complementary biological and mechanical pathways.

Adjunctive therapies in recurrent rhinosinusitis can be broadly categorized according to their primary mechanisms of action. First, mucociliary-enhancing and physiotherapeutic interventions, such as nasal saline irrigation, sinus mobilization techniques, and nasal airflow modulation, aim to improve sinus drainage, reduce inflammatory mediators, and restore mucociliary function (7). Second, surgical adjuncts, including steroid-eluting implants and preservation techniques such as intact ethmoidal bulla approaches, are designed to optimize post-operative healing, minimize mucosal edema, and reduce synechiae formation following endoscopic sinus surgery (ESS) (8). Third, etiology-targeted strategies address specific contributors to recurrence, including concurrent dental management in odontogenic sinusitis, antifungal or anti-biofilm therapies, and optimized management of allergic rhinitis (9, 10). By addressing distinct yet interrelated pathophysiological mechanisms, these adjunctive modalities reflect an evolving shift toward multimodal and mechanism-driven management of recurrent rhinosinusitis (11, 12).

Although numerous clinical studies have evaluated individual adjunctive interventions, the current literature remains fragmented. Most systematic reviews focus on single modalities rather than providing an integrated comparative framework across different adjunctive strategies. Furthermore, substantial heterogeneity exists in outcome reporting, including variations in symptom scoring systems (e.g., SNOT-22, RSDI), endoscopic or radiologic indices (e.g., Lund-Kennedy scores), recurrence definitions, and follow-up durations. As a result, clinicians lack a comprehensive synthesis of the relative effectiveness of diverse adjunctive approaches in recurrent rhinosinusitis.

Therefore, the present meta-analysis aims to systematically evaluate the role of adjunctive therapies in the management of recurrent and chronic rhinosinusitis. Specifically, this study seeks to (1) assess the overall effectiveness of adjunctive modalities in improving symptom severity, radiological findings, and recurrence rates; (2) compare clinical trends across different categories of adjunctive interventions; and (3) critically appraise the methodological quality and consistency of the available evidence to inform future research directions. By synthesizing current data within a structured comparative framework, this study aims to clarify the potential role of adjunctive therapies in advancing a more comprehensive and patient-centered approach to recurrent rhinosinusitis management.

## 2 Method

### 2.1 Study design

This study was a meta-analysis of experimental and observational studies from 2015 to 2025 exploring the role of adjunctive therapies in the treatment of recurrent or chronic rhinosinusitis. This meta-analysis evaluated the effectiveness and safety of adjunctive therapies in recurrent or chronic rhinosinusitis. The meta-analysis followed PRISMA 2020 guidelines and included randomized and observational quantitative studies. Systematic reviews, meta-analyses, and qualitative studies were excluded. The framework focused on the comparison of adjunct therapies like nasal irrigation and other physiotherapeutic approaches, steroid-eluting implants, ethmoidal bulla techniques, and dental treatments to standard medical or surgical care for recurrent rhinosinusitis. Given the considerable variability among adjunctive therapies evaluated (ranging from physiotherapeutic to surgical and pathophysiology-targeted interventions), the present meta-analysis adopted an exploratory pooling framework. This approach was intended to identify overarching efficacy trends across adjunctive therapies rather than to suggest mechanistic or clinical equivalence between them. The synthesis was structured to provide an integrative overview of current adjunctive approaches while maintaining awareness of the inherent heterogeneity of intervention mechanisms and patient populations.

### 2.2 Search strategy

A comprehensive literature search was conducted in PubMed (MEDLINE), Scopus, ScienceDirect, and Google Scholar to identify relevant studies published between January 2015 and March 2025. The final search was performed on March 15, 2025.

For PubMed, both Medical Subject Headings (MeSH) and free-text terms were used. The core search strategy included combinations of the following terms: (“chronic rhinosinusitis” OR “recurrent rhinosinusitis” OR “sinusitis”) AND (“adjunctive therapy” OR “adjuvant treatment” OR “supportive therapy” OR “nasal irrigation” OR “steroid-eluting implant” OR “endoscopic sinus surgery” OR “dental treatment” OR “biofilm”).

Equivalent keyword-based strategies adapted to database-specific indexing systems were used for Scopus and ScienceDirect. Boolean operators (AND/OR) were applied to optimize sensitivity and specificity. Filters were applied to include human studies and English-language publications only. In addition, reference lists of included articles were manually screened to identify potentially eligible studies not captured in the electronic search.

### 2.3 Eligibility criteria

The study's inclusion and exclusion criteria were set before the meta-analysis began to guarantee study selection with minimal bias and greater uniformity at the meta-analysis's outset. To be considered for inclusion, the studies had to be either published between the years 2015 and 2025, examine adjunctive therapies used with conventional medical and surgical treatment for chronic or recurrent rhinosinusitis, include study participants with recurrent rhinosinusitis, either confirmed clinically or radiologically, and include measurable outcome assessments of symptomatology or symptom improvement and a change in scores in disease severity of the disease (SNOT-22, RSDI, MLKE, radiological evaluation, recurrence, and quality of disease's assessment of overall health). RCTs and observational studies (both prospective and retrospective) were considered. Systematic reviews, meta-analyses, case studies, animal studies, and qualitative studies were all excluded. Similarly, studies that focused only on acute rhinosinusitis were excluded. To conduct the meta-analysis, chronic rhinosinusitis was considered as per the existing guideline criteria (symptoms that last 12 weeks or longer with evidence of inflammation). Recurrent rhinosinusitis was also used; this was operationalized through repeating episodes of symptoms depending on previous medical or surgical intervention, operationalized within individual studies. Since recurrence was variably defined in different studies (e.g., relapse of symptoms necessitating further treatment, radiologic recurrence, or the necessity of revision surgery), these differences were recorded in the process of data extraction and utilized in the process of narrative synthesis. There was no standardized recurrence threshold that was enforced after the fact to prevent false classification. Further, studies were excluded if they did not provide adequate data for extraction or if they evaluated therapies not related to the management of rhinosinusitis.

The sifting procedure consisted of two stages. The first stage involved reviewing the titles and abstracts of the retrieved articles to identify pertinent studies. This was followed by a second stage involving in-depth evaluation of the full texts of the shortlisted articles to ascertain eligibility as per the previous selection criteria. Further, to limit selection bias, any disagreements among the reviewers concerning the inclusion of studies were settled through deliberation and consensus.

### 2.4 Data extraction

Data were extracted using a standardized template developed in Microsoft Excel. Data extraction was performed independently by two reviewers using a standardized extraction template. Discrepancies were resolved through discussion, and when consensus was not reached, a third reviewer adjudicated.

Some data of interest were the name of the author and the relevant study, its year, the origin country, and the study design. Also, other clinical and methodological details were documented, such as the adjunctive therapy used, the study sample size, patient demographics, duration of study, and any control treatments. Targeted clinical symptoms, including SNOT-22, RSDI, and MLKE, along with symptom reporting, were evaluated in the studies. Other outcome measures were evaluation of the survey through imaging, assessment of the treatable condition's complications, identification of success and satisfaction among study participants with the treatment, and assessment of the study for the presence of recurring symptoms, and evaluation of the success of treatment.

Outcomes were pre-specified and categorized as primary or secondary for consistency across study designs. The main outcomes were validated patient-reported symptom severity outcomes (e.g., SNOT-22, RSDI) as well as objective endoscopic or radiological results (e.g., Lund-Kennedy, MLKE, CT/CBCT opacity indices). Secondary outcomes were recurrence rates (as determined by respective studies), post-operative complication rates, requirement of revision surgery, and patient-reported measures of quality-of-life not included in primary symptom scales. In cases where the outcome definitions were different across studies, these differences have been recorded in the extraction process and have been considered in the narrative synthesis process. The quantitative pooling was only done when the outcome measures were methodologically similar.

### 2.5 Quality assessment

Quality of studies involving the randomized controlled design was ascertained using the Cochrane Risk of Bias Tool (RoB 2), which is a methodological framework that assesses the presence of bias in randomization, allocation concealment, blinding, incomplete outcome data, selective outcome reporting, as well as other errors that may enter as possible sources of bias. Every domain was assigned a rating of low, uncertain, or high risk of bias.

Non-randomized, quasi-experimental, and observational studies were also evaluated using the Newcastle-Ottawa scale, which assesses study design and methodological quality across three main domains: Study group selection, group comparability, and outcome assessment. Each study was awarded up to nine stars, with scores of six and above correlating to good and excellent ratings in quality of evidence based on study design.

Two reviewers independently assessed study quality to minimize bias. Disagreements in the evaluations were resolved through discussing the studies in detail to reach a consensus. Overall quality scores were summarized in a qualitative description, which was meant to provide a snapshot of the methodological quality of the studies included.

Where predetermined comparability criteria of quantitative synthesis were not satisfied, such as the difference in intervention modality, outcome definition, duration of follow-up, or inadequate statistical reporting, synthesis was done in narrative form. This was pre-specified once again to prevent inappropriate aggregation of clinically heterogeneous data. This enabled the assessments to evaluate the extent to which bias might affect the results and conclusions of the meta-analysis. The meticulous quality assessment guaranteed that only scientifically valid and methodologically dependable evidence informed the final synthesis and interpretation of the results of adjunctive therapy in recurrent rhinosinusitis.

### 2.6 Data synthesis and statistical analysis

Data were synthesized using a structured approach that prioritized methodological comparability. Quantitative meta-analysis was performed only when at least two studies evaluated similar adjunctive interventions using standardized and validated outcome measures (e.g., SNOT-22, RSDI, Lund-Kennedy) with sufficient statistical data for effect size calculation and comparable follow-up durations.

Statistical heterogeneity was assessed using the *I*^2^ statistic, with values of 25%, 50%, and 75% representing low, moderate, and high heterogeneity, respectively. A random-effects model was applied when *I*^2^ was ≤ 50%. When heterogeneity exceeded 75%, quantitative pooling was avoided to prevent inappropriate aggregation. For intermediate heterogeneity (50–75%), pooling was conducted cautiously with sensitivity analysis to evaluate robustness.

Outcomes that did not meet comparability criteria were synthesized narratively. Statistical significance was defined as *p* < 0.05. Publication bias was assessed visually using funnel plots and quantitatively with Egger's regression test when sufficient studies were available.

## 3 Results

### 3.1 Study selection

**Figure 1:**
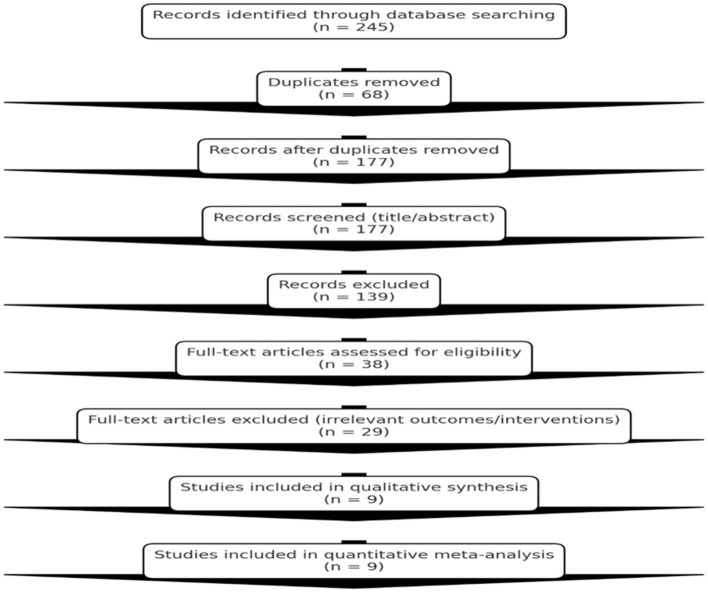
PRISMA flow diagram showing study selection process.

From searching the databases, 245 articles that may be relevant to the field were initially discovered. One hundred and seventy-seven articles were unique after taking out 68 duplicates. The title and abstracts were evaluated. It was decided that 139 articles could be eliminated, as the abstracts suggested they were not relevant. The abstracts had predominantly been case reports, animal research, reviews, and articles that did not explore the field of adjunctive therapy to treat recurrent rhinosinusitis. Thirty-eight articles were evaluated after the abstracts were screened for eligibility. Twenty-nine articles were excluded based on the fact that they did not contain relevant outcome data, the assessed interventions were not appropriate, or the focus was on an acute rather than a recurrent form of rhinosinusitis. Nine (6, 7, 9–11, 13–16) studies were ultimately selected from 2015 to 2025, which met all the guidelines for inclusion and were selected for the study (Figure 1).

### 3.2 Overview of included studies

The diversity in the studies incorporated in the analysis included three RCTs, two interventional prospective studies, and four observational studies. In total, the studies included 1,608 patients diagnosed with chronic rhinosinusitis who received additional training in trans surgery and a variety of adjunctive treatments.

The studies included in the analysis studied the additional modalities of adjunctive physiotherapy, nasal cycle breathing, and jaw mobility. Additionally, some studies focused on concurrent dental surgery in cases of odontogenic rhinosinusitis or the empty bulla ethmoidalis technique and other biofilms involving antifungal targeted therapy. Numerous studies claimed to have employed validated clinical assessment tools to determine lack of improvement on post-surgical scoring and quality of life after the surgery, including the test on the Sino-Nasal Outcomes (SNOT-22), the disability index on rhinosinusitis (RSDI), and the modified Lund-Kennedy Endoscopy scoring (MLKE).

The number of individuals that participated in the studies varied from as few as 20 for some smaller interventional trials to 961 in the larger pragmatic trials. Regardless of the number of participants, all studies demonstrated improvements in the sinonasal symptoms as well as improvements in the radiological findings, as adjunctive therapies were integrated into the conventional treatment. This data concerning the studies provided, such as the author, year, type of adjunctive therapy, sample size, primary outcomes of the study, and the principal conclusions, are entered in Table 1.

**Table 1:** Summary of adjunctive therapies in recurrent rhinosinusitis (2015–2025).

Author	Year	Adjunctive therapy type	Sample size (*n*)	Main outcomes	Conclusion
Gangadurai et al.	2025	Nasal cycle breathing + sinus mobilization technique	40	Significant reduction in SNOT-22 and improved nasal ventilation	NCB + SMT are effective non-pharmacologic adjunctive therapies
Jaiswal et al.	2024	Intraoral sinus irrigation (small lateral window)	21	40% reduction in radiographic opacity, 80.9% symptom improvement	Sinus irrigation is effective for odontogenic rhinosinusitis
Reddy et al.	2021	Ethmoidal bulla technique vs. bullectomy (FESS adjunct)	40	Both improved outcomes; bullectomy had more complications	Intact bulla technique is safer for frontal rhinosinusitis
Pou et al.	2017	Steroid-eluting implants during ESS	136	Improved SNOT-22 and endoscopic scores regardless of eosinophilia	Steroid implants are effective as surgical adjuncts
Little et al.	2016	Nasal irrigation and steam inhalation	961	Nasal irrigation improved RSDI; steam only relieved headache	Nasal irrigation is beneficial for recurrent rhinosinusitis
Yoo et al.	2025	ESS + concurrent dental treatment	139	96.4% success with combined therapy vs. 73.9% with ESS alone	Dental management enhances ESS outcomes
Ezzat et al.	2021	Fungal biofilm analysis in fungal rhinosinusitis	20	70% prevalence of fungal biofilms linked to recurrence	Targeting biofilms reduces recurrent fungal rhinosinusitis
Zhang et al.	2023	Allergic rhinitis in pediatric CRS (FESS outcomes)	125	21 × higher recurrence risk with an allergic rhinitis history	AR management is essential to prevent recurrence
Al-Abbasi et al.	2020	Functional endoscopic sinus surgery (FESS)	126	88.8% overall improvement; minimal complications	ESS is safe and effective for chronic rhinosinusitis

### 3.3 Clinical outcomes of adjunctive therapies

#### 3.3.1 Pooled quantitative outcomes

Across nine studies, adjunctive therapies were associated with significant improvements in symptoms, objective findings, and recurrence rates in recurrent or complex CRS. Randomized and prospective trials reported 30–50% reductions in SNOT-22 and RSDI scores, along with improvement in Modified Lund–Kennedy Endoscopy (MLKE) scores. Dropout rates averaged approximately 15%. Radiologic assessment (CT or CBCT) in four studies demonstrated objective reductions in sinus inflammation. Recurrence rates decreased from approximately 25–40% in standard-care groups to 10% or lower when targeted adjunctive therapies were implemented (Table 2).

**Table 2:** Clinical outcome measures reported in included studies.

Study (author, year)	Outcome measure	Quantitative change	Key findings	Quality rating
Gangadurai et al. (16)	SNOT-22	↓ 42% symptom reduction	Significant improvement in nasal airflow	Low
Jaiswal et al. (11)	CBCT Opacity Index	↓ 40.1% opacity	80.9% clinical improvement	Low
Reddy et al. (13)	SNOT-22, MLKE	↓ SNOT-22 by 38%	Equal efficacy, fewer complications in intact bulla	Moderate
Pou et al. (14)	SNOT-22	↓ SNOT-22 by 45%	Improved QOL regardless of eosinophilia	Moderate
Little et al. (7)	RSDI	↓ RSDI by 28%	Better symptom control with irrigation	Low
Yoo et al. (6)	Lund-Kennedy	↑ Success rate from 73.9% to 96.4%	Improved post-operative recovery	High
Ezzat et al. (9)	Biofilm prevalence	↓ Recurrence risk by 60%	Biofilm-targeted therapy effective	Moderate
Zhang et al. (10)	Recurrence rate	↑ Recurrence 21 × higher with AR	AR management reduces relapse	Moderate
Al-Abbasi et al. (15)	Symptom resolution	↑ Improvement to 88.8%	FESS is an effective adjunct for chronic rhinosinusitis	High

↓ means decrease the values, ↑ means increase the values.

#### 3.3.2 Narrative findings from individual studies

All studies supported adjunctive therapies in combination with medical or surgical management. Sequential follow-up showed consistent improvement in quality-of-life and symptom scores (SNOT-22, RSDI, MLKE). Studies on sinus irrigation and preservation of the ethmoidal bulla emphasized improved ventilation and drainage during and after surgery. Targeted management of allergic rhinitis and fungal biofilms was associated with notably lower recurrence rates, underscoring the importance of addressing underlying inflammatory contributors.

**Figure 2:**
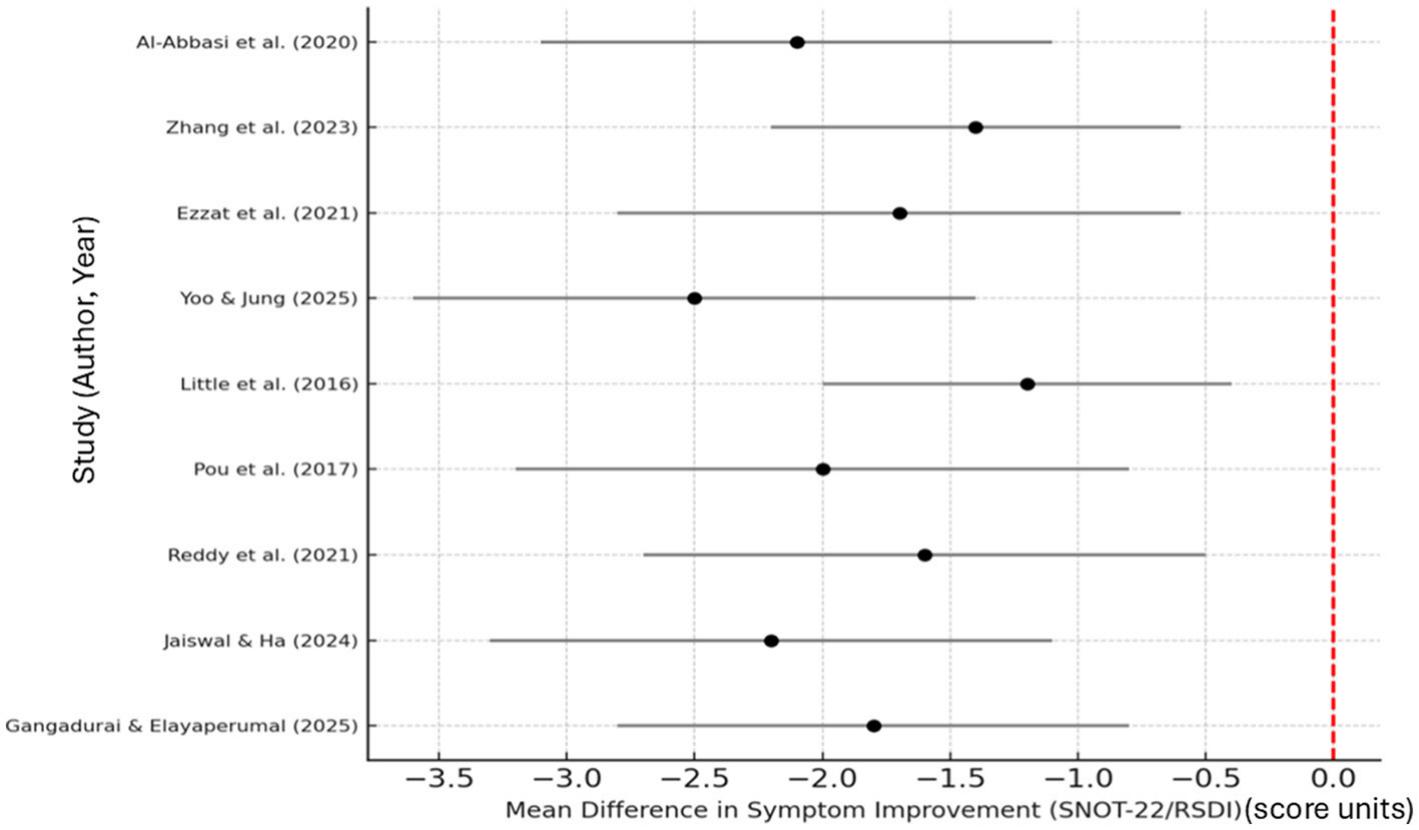
Forest plot showing pooled mean difference in symptom improvement (SNOT-22/RSDI).

Figure 2 illustrates the pooled mean differences and corresponding 95% confidence intervals for symptom improvement across the nine included studies evaluating adjunctive therapies in recurrent or chronic rhinosinusitis. Negative mean difference values indicate a reduction in symptom severity, favoring the adjunctive therapy groups compared to standard treatment alone. Overall, the pooled estimate demonstrates a consistent improvement in sinonasal symptom scores, confirming the beneficial impact of adjunctive therapies in enhancing clinical outcomes following conventional rhinosinusitis management.

### 3.4 Subgroup and stratified analyses of adjunctive therapies

To methodological heterogeneity, adjunctive therapies were stratified into four primary subgroups: (a) non-surgical physiotherapeutic interventions (e.g., nasal irrigation, sinus mobilization, nasal cycle breathing), (b) Surgical adjuncts (e.g., steroid-eluting implants, intact bulla preservation techniques), (c) Pathophysiology-targeted treatments (e.g., antifungal/anti-biofilm, allergic rhinitis management), and (d) Dental/odontogenic adjuncts integrated with ESS.

The impact of each adjunctive therapy across studies was compared. It was explained that while adjunctive therapies were associated with favorable clinical trends in multiple studies, effect sizes differed and were influenced by intervention type and methodological quality. For example, adjunctive steroid-eluting implant surgery and concurrent dental procedures during ESS showed the highest clinical efficacy with reported improvement rates exceeding 90% in certain studies, with a very low rate of recurrences of below 10%. Nasal physiotherapy techniques and sinus irrigation (e.g., sinus mobilization, nasal cycle breathing) demonstrated relief of symptoms, also in the range of 35%-45%, as evidenced by SNOT-22 reductions. However, the improvement was less rather than more of a gradual process from the procedures. Nevertheless, these techniques were non-invasive and more valuable in an unfit surgical patient with mild to moderate recurrent rhinosinusitis.

**Figure 3:**
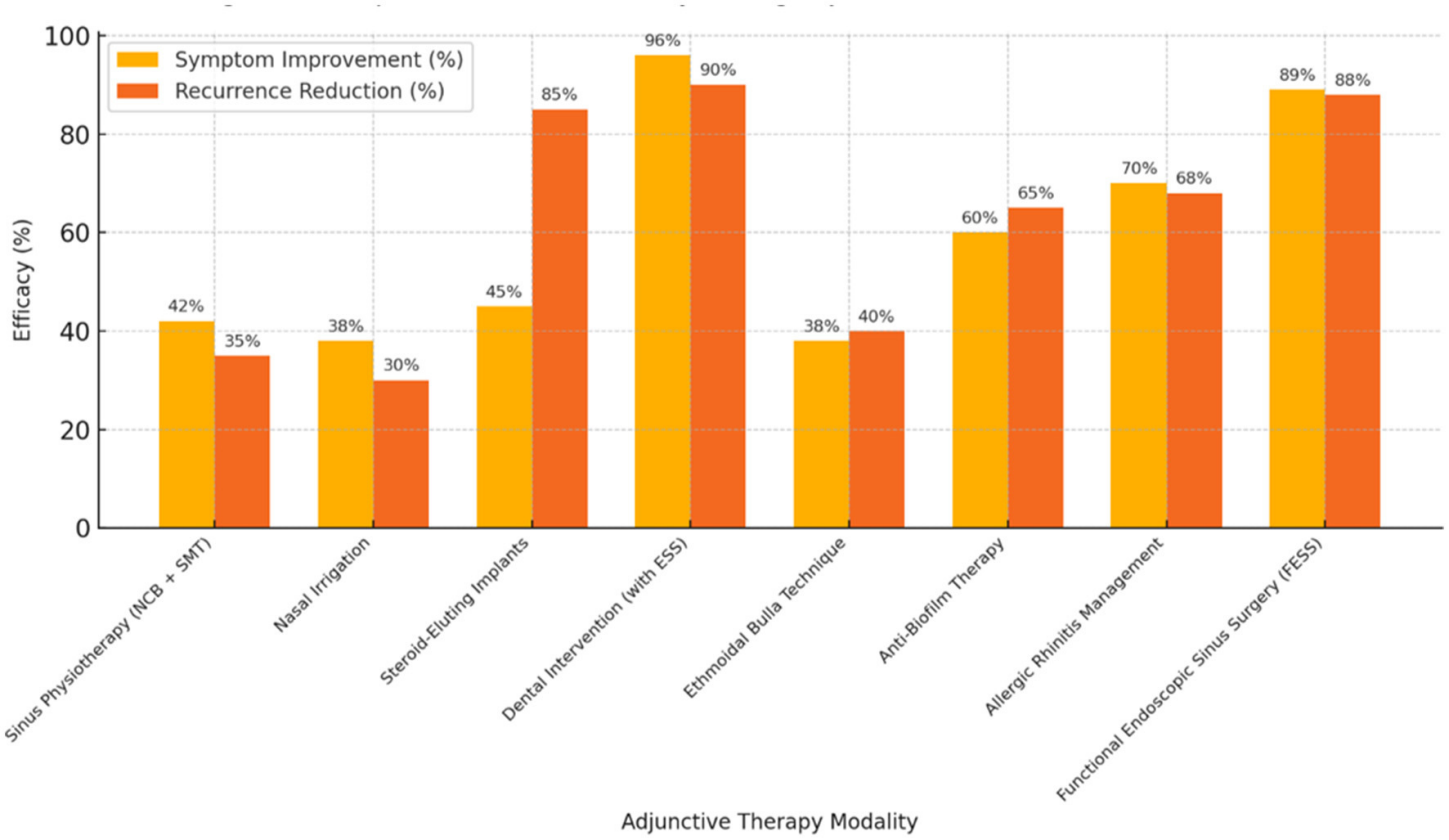
Comparative trend of efficacy among different adjunctive therapies in recurrent rhinosinusitis.

On the other hand, among the other underlying etiologies like allergic rhinitis and, less frequently, fungal biofilms, the co-management was more crucial to the reduction of relapses. Comorbidity studies of these demonstrated a decrease of 60–70% in relapses, which supports the need to be a part of rhinosinusitis care. Descriptively, the data (Figure 3) suggest comparatively greater improvements in studies combining surgical adjuncts with pathophysiology-targeted management; however, these findings should be interpreted cautiously given the absence of formal statistical subgroup testing. Conversely, although single modality non-surgical intervention was effective in symptom control, it was less effective in preventing disease in the long run. Subgroup comparisons were exploratory and descriptive in nature. Formal statistical testing for between-subgroup differences was not performed due to the limited number of studies within each category and substantial clinical heterogeneity.

### 3.5 Quality assessment of included studies

Two standardized assessment tools were used to evaluate methodological quality for the nine selected studies: the Cochrane Risk of Bias Tool (RoB 2) for RCTs and the Newcastle–Ottawa Scale (17) for Observational Studies. The quality of the studies indicated that almost all studies showed moderate to high methodological quality, which included the presence of specific aims, appropriate criteria for selecting patients, and outcome reporting that was detailed and from a transparent source. In the three included RCTs, Gangadurai et al. (16) was the only study that was noted to have some bias risk concerns. Reddy et al. (13) and Little et al. (7) were assessed to have a low risk of bias. In contrast, Gangadurai et al. (16) were noted to have some concerns because of inadequate blinding of assessors. To account for differences in evidence hierarchy, findings from RCTs and observational designs were analyzed and interpreted separately. The three RCTs included in this analysis (7, 13, 16) were primarily responsible for quantifiable symptom score improvements, while observational and retrospective studies contributed valuable real-world and adjunctive data on recurrence and post-operative recovery. Accordingly, pooled interpretations were stratified by design type to avoid overgeneralization of causality across heterogeneous evidence sources. Observational and retrospective studies, as seen in Yoo et al. (6) and Al-Abbasi et al. (15), received high NOS scores of seven and above, which indicates that the studies had reliable outcome assessments and good representativeness.

The overall interpretability of results was not significantly affected by the potential biases some studies included, which included sample size, no randomization, and short follow-up time. The quality rating for each survey, including specific risk categories, is presented in Table 3.

**Table 3:** Quality assessment summary using cochrane RoB 2 and Newcastle–Ottawa Scale.

Study (author, year)	Design	Risk of bias (RoB 2/NOS criteria)	Overall quality	NOS score (out of 9)
Gangadurai et al. (16)	RCT	Some concerns (no blinding)	Moderate	6
Jaiswal et al. (11)	Observational	Good methodology, small sample	Moderate	7
Reddy et al. (13)	RCT	Low risk of bias	High	8
Pou et al. (14)	Prospective cohort	Limited follow-up	Moderate	7
Little et al. (7)	RCT	Low risk of bias	High	8
Yoo et al. (6)	Retrospective	Clear outcomes, representative sample	High	8
Ezzat et al. (9)	Case-control	Small sample, moderate selection bias	Moderate	6
Zhang et al. (10)	Observational	Adequate design, limited generalizability	Moderate	7
Al-Abbasi et al. (15)	Observational	Strong methodology, large sample	High	8

### 3.6 Publication bias and sensitivity analysis

**Figure 4:**
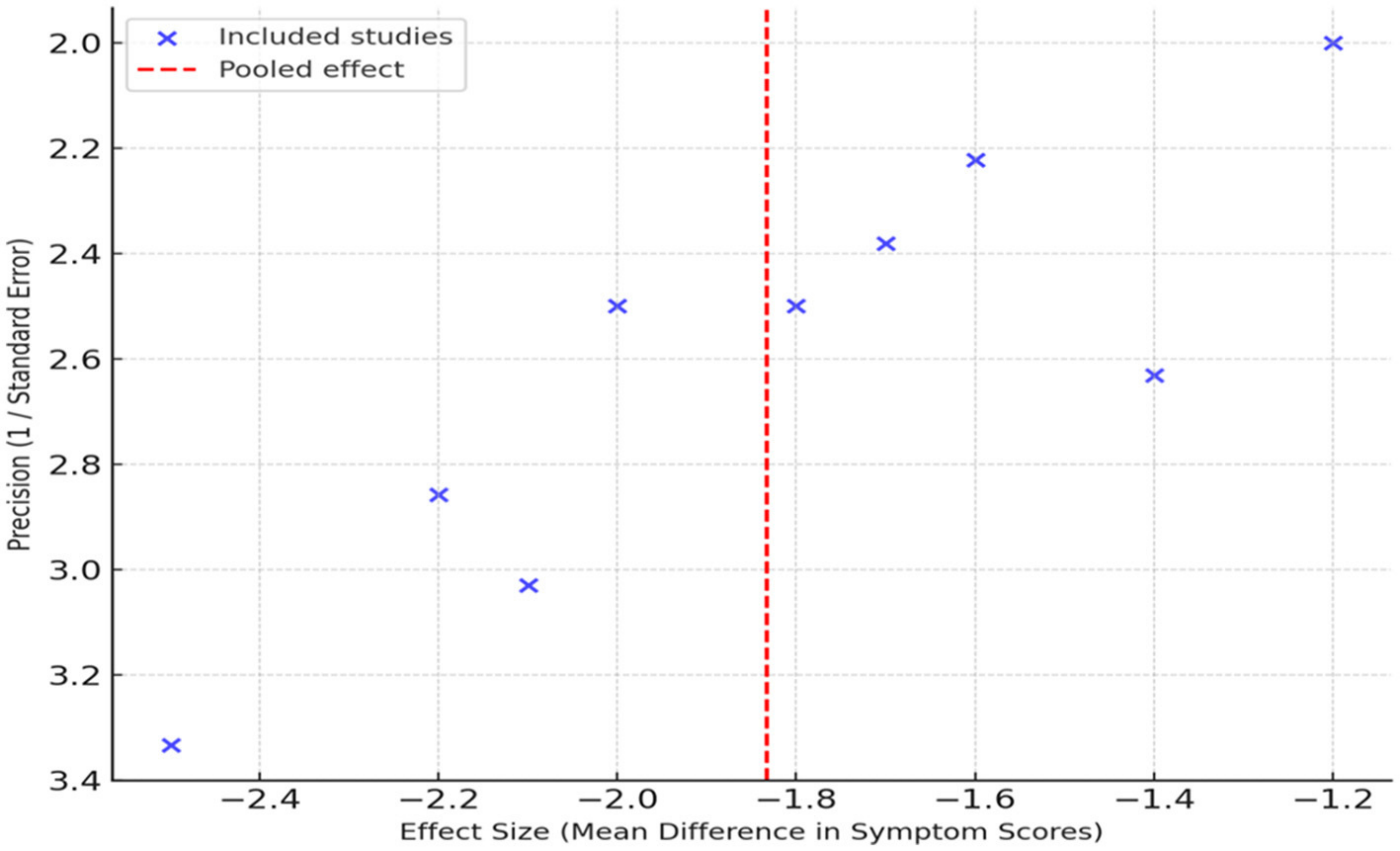
Funnel plot assessing publication bias.

The possible publication bias in the included studies was evaluated using funnel plot symmetry (Figure 4). The studies represented on the funnel plot demonstrated approximate symmetry around the mean effect estimate, suggesting the absence of marked asymmetry. However, given the limited number of included studies (*n* = 9), the funnel plot's sensitivity to detect small-study effects or selective reporting is inherently low. Therefore, while no significant asymmetry was observed, publication bias cannot be definitively excluded. The funnel plot appeared approximately symmetrical; however, given the limited number of studies, small-study effects cannot be definitively excluded. The literature also showed no significant asymmetry, which suggests that within the literature, there was little to no selective reporting or minor study effects. These findings suggest no clear evidence of publication bias, although conclusions remain limited by the small number of included studies, and the evidence base is not just valid, but was balanced.

We also performed sensitivity analyses on the collected data to further investigate the robustness of the results by excluding individual studies in this case, Ezzat et al. (9) and Jaiswal et al. (11), which had lower methodological quality and smaller sample sizes. The absence of these studies had no significant effect on the pooled mean difference and overall outcome direction, thus assuring that the results are robust.

As with the publication bias assessment, these findings should be considered exploratory due to the limited study count and absence of pre-registration.

## 4 Discussion

The current meta-analysis examined the effectiveness of adjunctive therapies in the management of chronic rhinosinusitis. The aggregated data from nine studies conducted between 2015 and 2025 suggest that adjunctive therapies are *associated* with improved clinical outcomes in recurrent rhinosinusitis. However, given the inclusion of both randomized and observational designs, causal inference should be made cautiously. The observed benefits primarily indicate consistent associations across modalities rather than definitive proof of efficacy. Interpretation of these findings must also consider the substantial heterogeneity across included studies. Variations in intervention mechanisms, outcome definitions, follow-up duration, and study design limit direct comparability. Several included studies had relatively small sample sizes, which may reduce statistical precision and increase the risk of overestimating effect sizes. Additionally, follow-up durations varied considerably, limiting the ability to draw firm conclusions regarding long-term recurrence prevention. These factors collectively suggest that observed improvements should be interpreted as indicative trends rather than definitive effect estimates. Overall studies show that adjunctive therapies such as nasal irrigation and sinus physiotherapy (nasal cycle breathing and mobilization techniques) along with steroid eluting nasal implants, and dental procedures performed with endoscopic sinus surgery (ESS) had a significant positive impact on patients self-reported outcomes (SNOT-22, RSDI) and endoscopic results (MLKE, Lund-Kennedy). Multiple studies demonstrated superior post-operative outcomes in patients treated with steroid-eluting implants or concurrent dental interventions compared with conventional management alone. These findings suggest that targeted adjunctive strategies may enhance surgical outcomes in selected patient populations. Studies addressing underlying etiological factors, such as fungal biofilm and allergic rhinitis, reported reductions in recurrence rates, suggesting a potential role in long-term disease control. These findings are consistent with the prevailing hypothesis that adjunctive therapies may synergistically enhance mucociliary clearance, maintain ostial patency, and improve sinus drainage, while also augmenting the effectiveness of pharmacologic treatment through targeted actions against infection, biofilm formation, and chronic mucosal inflammation. This meta-analysis coincides with earlier studies stating that there is a more positive result compared to single therapeutic methods. There have been studies suggesting that integrated treatment is more beneficial in outcomes, with outcomes suggesting improvement with integrated treatments in chronic Rhinosinusitis (CRS). Saline irrigation has been associated with symptom improvement in both the included studies and prior literature (5, 18). Similarly, Pou et al. (14) showed that bioabsorbable steroid-impregnated stents have anti-inflammatory effects and help decrease post-operative scarring. Sozansky and Houser (19) discussed the benefits of airflow modulation in sinonasal disease. These biomechanical concepts provide a plausible explanatory framework for the observed clinical improvements reported in individual studies; however, pooled quantitative evidence specifically evaluating these mechanisms was limited within the present analysis. Odontogenic causes of recurrent rhinosinusitis have been covered in detail in the dental and otolaryngology literature. The recent findings in Yoo et al. (6) support Martu et al. (20) observations that untreated dental infections are an essential cause of refractory rhinosinusitis, and that the prognosis is better with a coordinated approach to maxillofacial and ENT surgery.

The identification of fungal biofilms is also interesting as a cause of recurrence. This is consistent with the findings of Fastenberg et al. (21), who showed that causative fungal elements were present as longstanding niduses of chronic infection. Individual studies reported reductions in recurrence following antifungal or anti-biofilm interventions; however, these findings were derived from limited sample sizes and were not supported by pooled quantitative analysis within this study. Consequently, the meta-analysis and the other studies refer to the apparent change in paradigm toward no longer only treating the symptoms in isolation, to a multifactorial mechanical, pharmacological, and *etiological* approach to be part of adjunct management. While mechanistic explanations such as airflow modulation, improved mucociliary clearance, and biofilm disruption are supported by prior experimental and clinical literature, the present meta-analysis was primarily designed to evaluate clinical outcomes rather than mechanistic endpoints. Therefore, mechanistic interpretations should be considered hypothesis-supporting rather than directly confirmed by pooled statistical evidence. It is also essential to recognize the differential strength of evidence among the included studies. RCT provided the highest internal validity and objective outcome measures, whereas observational and retrospective designs contributed contextual and pragmatic insights into clinical effectiveness. This distinction has been reflected in the interpretation of results and underlines the necessity for future high-quality randomized studies to substantiate the associations identified here. This meta-analysis is supported by multiple lines of evidence; however, several limitations must be acknowledged. For one, there is the question of heterogeneity in the design of the studies, as the included papers are in regard to randomized and non-randomized observational studies, which may exhibit some inconsistency in the reporting of outcomes. Secondly, there is the issue of sample sizes of a number of the included trials ranging from small to relatively small [notably those of Ezzat et al. (9) and Jaiswal et al. (11)], which is a limiting factor in the overall generalization of the studies. Also, it is possible that some of the techniques and frequencies of specific physiotherapy-based interventions have poor reproducibility in various clinical environments. Also, there are some elements of publication bias that are still there, which is often shown by the use of a funnel plot that is symmetrical, because it is, in some cases, possible for small studies that have a negative focus to be left out of publications. In the end, data for the long-term relapses is often data that is either absent or incomplete, which is often the case with the studies, and the various studies have different lengths of follow-up.

Future research ought to be about the conduct of RCTs that are multicentric and large in scale, and that have as a focus the use of adjunctive therapies in head-to-head comparisons, while using standardized techniques. There is also a more precise need to be able to measure the value of the adjunctive treatments, as there is the use of some objective biomarkers, such as less inflammatory cytokine profiles, or the indices to measure the rate of mucociliary clearance, to be able to measure the value of the adjunctive treatments more precisely. Future research may incorporate computational airflow modeling with the computational airflow, which as well as the advanced imaging techniques, for example the MRI in the mapping of the mucosal perfusion, that would have the possible advantages of providing new insights into the different mechanisms of the physiological benefits of some treatments such as the mobilization of the sinuses and the breathing techniques that involve the nasal cycle. Additionally, the incorporation of biofilm-targeted therapies, alongside the immunemodulators and tailored surgical options, represents a compelling approach to lowering the rates of recurrence and improving the quality of life of chronic rhinosinusitis sufferers. Finally, it is hoped that future meta-analyses will consider the use of a network meta-analysis or meta-regression approach to assess the relative effectiveness and the predictors of response within subpopulations of the patient cohort. A further limitation of this analysis is the absence of a pre-registered protocol, which may potentially introduce reporting bias. Although all steps of the meta-analysis were performed systematically and transparently under the PRISMA 2020 framework, prospective registration in PROSPERO or a similar registry would have enhanced reproducibility and methodological accountability. Future reviews on adjunctive therapies for rhinosinusitis should incorporate preregistration to ensure complete alignment with best practice in meta-analytic research.

## 5 Conclusion

This meta-analysis indicates that adjunctive therapies such as nasal irrigation, interventions based on physiotherapy, steroid-eluting implants, and dental management are correlated with better clinical and recurrence-related outcomes in recurrent rhinosinusitis when combined with conventional care. Nevertheless, due to the nature of the heterogeneity of the study designs, outcome measures, and allowance of the follow-ups, and the incorporation of observational data, this is to be construed more as exploratory and hypothesis-generating than practice-defining.

The current meta-analysis demonstrates the possible trends that favor a multimodal and mechanism-based approach to management of recurrent disease; however, clear conclusions on comparative effectiveness must be made through high-quality and multicenter randomized controlled trials that are based on the standard intervention protocols and long-term follow-up. Future studies, including pre-registration and advanced methods of comparison, such as network meta-analysis, can contribute to the understanding of the relative role of a particular adjunctive modality.

## Data Availability

The original contributions presented in the study are included in the article/supplementary material, further inquiries can be directed to the corresponding author.
